# A Meta-Analysis of the Global Stillbirth Rates during the COVID-19 Pandemic

**DOI:** 10.3390/jcm12237219

**Published:** 2023-11-21

**Authors:** Manoj Mohan, Kwabena Appiah-Sakyi, Ashok Oliparambil, Abdul Kareem Pullattayil, Stephen W. Lindow, Badreldeen Ahmed, Justin C. Konje

**Affiliations:** 1 Department Obstetrics and Gynecology, AsterDM Healthcare, Doha P.O. Box 8703, Qatar; manojmohan@doctors.org.uk; 2 Obstetrics and Gynecology, Britannia Medical Centre, Community 22, Tema, Ghana; kasakyi@hotmail.com; 3 Physician Obstetrics and Gynecology, Sidra Medicine, Doha, Qatar; asholip@gmail.com; 4 Sidra Medicine, Doha P.O. Box 26999, Qatar; apullattayil@gmail.com; 5 Masters Projects, Coombe Women and Infants University Hospital, D08 XW7X Dublin, Ireland; slindow@coombe.ie; 6 Feto Maternal Centre, Doha P.O. Box 34181, Qatar; profbadreldeen@hotmail.com; 7 Obstetrics and Gynaecology, Qatar University and Weill Cornell Medicine, Ar-Rayyan P.O. Box 24144, Qatar; 8 Obstetrics and Gynaecology, Department of Health Sciences, University of Leicester, Leicester LE2 7LX, UK; 9 Obstetrics and Gynaecology, Weill Cornell Medicine, AL-Rayyan P.O. Box 24144, Qatar

**Keywords:** SARS, COVID-19, stillbirth, meta-analysis, pre- and post-pandemic

## Abstract

COVID-19 has been shown to have variable adverse effects on pregnancy. Reported data on stillbirth rates during the pandemic have, however, been inconsistent—some reporting a rise and others no change. Knowing the precise impact of COVID-19 on stillbirths should help with the planning and delivery of antenatal care. Our aim was, therefore, to undertake a meta-analysis to determine the impact of COVID-19 on the stillbirth rate. Databases searched included PubMed, Embase, Cochrane Library, ClinicalTrials.gov, and Web of Science, with no language restriction. Publications with stillbirth data on women with COVID-19, comparing stillbirth rates in COVID-19 and non-COVID-19 women, as well as comparisons before and during the pandemic, were included. Two independent reviewers extracted data separately and then compared them to ensure the accuracy of extraction and synthesis. Where data were incomplete, authors were contacted for additional information, which was included if provided. The main outcome measures were (1) stillbirth (SB) rate in pregnant women with COVID-19, (2) stillbirth rates in pregnant women with and without COVID-19 during the same period, and (3) population stillbirth rates in pre-pandemic and pandemic periods. A total of 29 studies were included in the meta-analysis; from 17 of these, the SB rate was 7 per 1000 in women with COVID-19. This rate was much higher (34/1000) in low- and middle-income countries. The odds ratio of stillbirth in COVID-19 compared to non-COVID-19 pregnant women was 1.89. However, there was no significant difference in population SB between the pre-pandemic and pandemic periods. Stillbirths are an ongoing global concern, and there is evidence that the rate has increased during the COVID-19 pandemic, but mostly in low- and middle-income countries. A major factor for this is possibly access to healthcare during the pandemic. Attention should be focused on education and the provision of high-quality maternity care, such as face-to-face consultation (taking all the preventative precautions) or remote appointments where appropriate.

## 1. Introduction

The global burden of stillbirth(SB) continues [1], with an estimated two million every year. COVID-19 has an adverse effect on pregnancies [2,3], but there have been conflicting reports on increasing SB rates during the pandemic [4,5,6,7]. 

A population study from two Philadelphia Hospitals in the USA [8] did not detect any stillbirth changes with COVID-19, but a study from Nepal [9] showed a higher rate of stillbirth during the COVID-19 lockdown and an associated increase in neonatal deaths [10]. Similarly, pregnancy outcomes from St Georges University Hospital, London, UK [6], also showed an increased SB rate. However, reports from the Spanish population study [11], hospital episode statistics from England [12], and a case–control study from Lady Hardinge Medical College, India [7], showed no change. The population study from Nepal [9] suggested that the increase may be due to decreased access to high-quality healthcare rather than a direct effect of the viral illness on pregnant women. While there are currently published protocols about ongoing studies to determine the adverse effects in pregnancy, such as “*the COVID-19 in pregnancy in Scotland (COPS) study*” [13], published studies, however, do not provide a clear overview of the impact on stillbirths during the pandemic.

We, therefore, undertook a meta-analysis of published studies on the impact of COVID-19 on stillbirths with the hope of providing more robust data on overall population rates and comparison between COVID-19 and non-COVID-19 pregnancies, as well as pandemic and pre-pandemic periods. Furthermore, we also examined the contribution of the income status [14] of countries to SB rates. 

## 2. Method

### 2.1. Search

**Figure 1:**
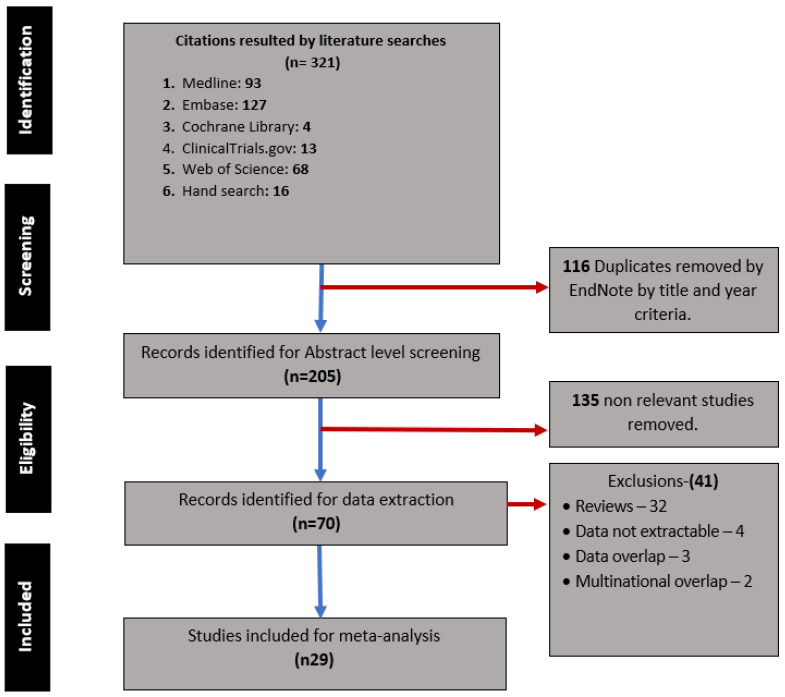
PRISMA Flowchart.

A systematic search and screening were conducted by two independent reviewers (MM and KA) with the support of AP on Embase, PubMed, Cochrane Library, clinicaltrials.gov (accessed on 20 March 2022), and Web of Science. The search terms used were “COVID-19 Pregnancy, Stillbirth, Intrauterine fetal death/demise” (Supplementary Materials S1 Search Strategy). The initial screening was conducted using titles and abstracts (MM and KA), and then further screening was carried out using pre-agreed eligibility criteria. All studies from similar countries of origin were checked to avoid data duplication, and five studies were excluded [6,15,16,17,18], as shown in Figure 1. 

### 2.2. Inclusion Criteria

We included studies that published data on stillbirths in pregnant women with COVID-19 and studies that compared population (women with and without COVID-19) SB rates before and during the COVID-19 pandemic, with no language restrictions. As there is variation in the definition of SB with regard to the gestational age (from one country to another with cut-offs varying from 20 to 28 weeks of gestation), we used the definitions in each of the studies. We included observational prospective and retrospective studies, case series, and letters with observational data. We excluded case reports and studies on multinational registries that overlapped across countries to avoid duplication.

### 2.3. Outcome Measures

Three separate outcomes related to SB were assessed.

1. SB rate in pregnant women with COVID-19;
2. SB rates in pregnant women with and without COVID-19 during the same time period;
3. Population SB rates in pre-pandemic and pandemic periods.

### 2.4. Risk of Bias

Two independent reviewers (MM and AO) completed the quality assessment of each identified publication separately, and uncertainty or disagreements were resolved by consensus with further review by SWL and JCK. 

We used the Newcastle–Ottawa quality assessment scale (NOS) [19] to define the eligibility of the observational epidemiology studies. This tool, based on 8 items, has 3 categories—the selection of the study groups, the comparability of the groups, and the ascertainment of either the exposure or outcome of interest for case–control or cohort studies. As per the tool, a study could be given a maximum of one star for each numbered item within the selection and outcome categories and a maximum of two stars within the comparability category. Studies with total scores of 0–3 stars (red color), 4–6 stars (yellow color), and 7–9 stars (green color) are classified as studies with high, moderate, or low risk of bias, respectively. 

### 2.5. Data Extraction

Two independent reviewers (MM and AO) extracted the data onto a pre-defined Microsoft Excel spreadsheet; this was done after the exclusion of possible duplicated data. We cross-checked that studies from the same counties were from different hospitals and population groups. As shown in the PRISMA flow chart, we removed studies suspected duplication (Figure 1). Two of the included studies had data queries. Therefore, we contacted the authors for details of the information and received clarification and, thus, had that data in this analysis [20,21]. 

### 2.6. Meta-Analysis

The Comprehensive Meta-Analysis version 3 (CMA version 3) tool [22] was used for analysis. We estimated the SB rate based on the number of stillbirths and deliveries from each included study. The studies were pooled using a mixed-effect meta-analysis with a 95% confidence interval. In the mixed effect analysis, a random effects model was used to combine studies within each subgroup. A fixed effect model was used to combine subgroups to generate an overall effect. The study-to-study variance (Tau-squared), as expected, was not assumed to be the same for all subgroups; this value was computed within subgroups and not pooled across subgroups. We present the pooled overall event rate along with subgroup event rates. When the two groups were compared, we used the SB numbers and total pregnancies from each study, both in the SB and comparison groups and a pooled estimate (odds ratio) with a 95% confidence interval was provided. 

### 2.7. Heterogeneity

We took into consideration the weighted pooled effect size and considered how much the effects varied from study to study. The relevant statistics are given with a Q-value with the degrees of freedom and a *p*-value. For statistical heterogeneity and variance interpretation [22], we have also provided the I-squared and Tau-squared. 

## 3. Results

The 29 studies included in this meta-analysis were both from high-income (HIC) (United States of America, United Kingdom, Ireland, Sweden, Spain, Italy, Israel, Kuwait, and French Guiana) and low- and middle-income countries [14] (LMIC) (Oman, Botswana, Peru, India, and Nepal). Seventeen studies showed a low risk of bias, and twelve were of moderate risk of bias. A funnel plot of the precision by rate was used to depict the publication bias in the included observation studies (Supplementary Materials S2).

**Figure 2:**
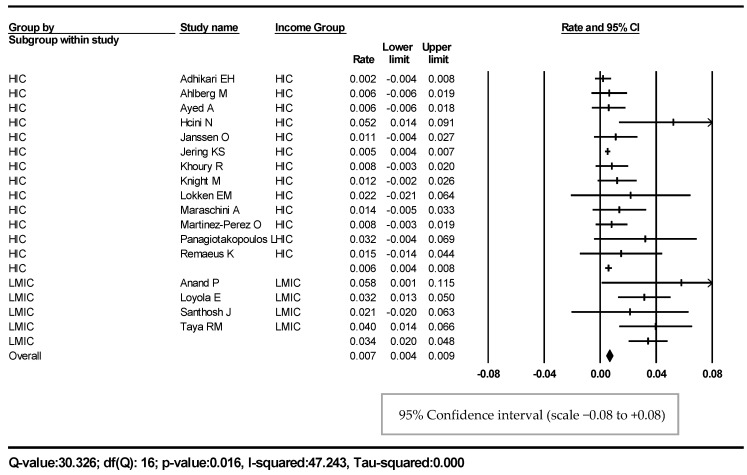
Forest plot, outcome one. SB rate in pregnant women with COVID-19.

For the first outcome, we included 17 studies [23,24,25,26,27,28,29,30,31,32,33,34,35,36,37,38,39] with 9476 COVID-19 pregnancies and 95 stillbirths (Figure 2). The overall pooled SB rate was 0.7% (95% CI 0.4–0.9%). 

A subgroup analysis showed the SB rate in the HIC group to be 0.6% (95% CI 0.4–0.8%), compared to that of 3.4% (95% CI 2–4.4%) in the LMIC group (Figure 2).

We performed a sensitivity analysis to explore the robustness of our results from the 17 included studies. We conducted this firstly by removing five of the included studies from the USA [23,27,29,31,34], as the USA was the largest representing country, and this showed a similar pooled SB rate of 0.8% (95% CI 0.5–1.2%). A further sensitivity analysis was done by including only studies with a low risk of bias [23,24,26,27,28,30], and the pooled rate was 0.6% (95% CI 0.2–1.1%). This sensitivity analysis confirmed the robustness of the results from the 17 studies.

**Figure 3:**
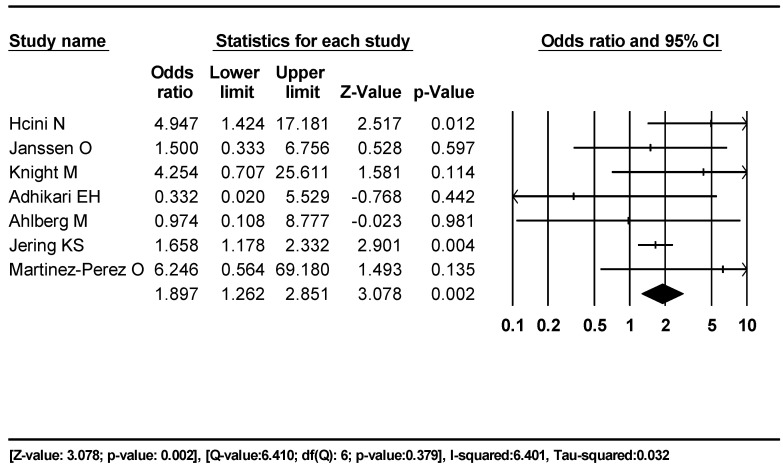
Forest plot, outcome two. SB rates in pregnant women with and without COVID-19 during the same time period.

For the second outcome of SB rates in concurrent COVID-19 and non-COVID-19 pregnancies, we included seven studies [23,24,26,27,28,33,40]. All were from HIC, and therefore, we used a random effect model for comparison. There were 7587 pregnancies with 49 stillbirths and 40,7139 pregnancies with 1330 stillbirths in women with and without COVID-19, respectively (Figure 3). The odds ratio of SB in COVID-19 pregnancies was 1.897 (95% CI 1.262–2.851) compared to non-COVID-19 pregnancies.

**Figure 4:**
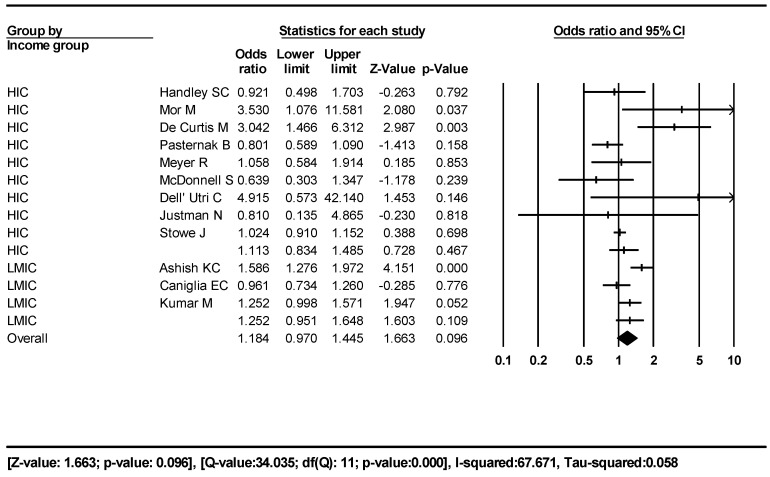
Forest plot, outcome three. Population SB rates in pre-pandemic and pandemic periods.

For our third outcome, SB rates in pre-pandemic and pandemic periods, we included 12 studies [7,8,9,12,20,21,41,42,43,44,45,46]—184,288 pregnancies and 1038 stillbirths and 292,159 pregnancies and 1517 stillbirths in the pre-pandemic and pandemic periods, respectively. We used a mixed-effect model similar to outcome one for this analysis, and the results are shown in Figure 4. The odds ratio of SB during the pandemic period was 1.184 (95% CI 0.970–1.445) compared to the non-pandemic period.

The subgroup analysis showed that HIC had an OR of 1.113 (95% CI 0.834–1.485) versus 1.252 (95% CI 0.951–1.648) for LMIC (Figure 4).

## 4. Discussion

In this meta-analysis, the overall SB rate in COVID-19 pregnancies was 0.7% (95% CI 0.4–0.9%). It was 0.66% (95% CI 0.4–0.8%) in the HIC group of countries compared to 3.44% (95% CI 2.0–4.8%) in the LMIC. The risk of SB was much higher in COVID-19 compared to non-COVID-19 pregnancies, but the overall population SB rate was not different between the pre-pandemic and pandemic periods.

There were significant variations in SB rates (1.4–32.2 per 1000 total births) across the world [47], with a much lower rate in HIC [48]. In LMIC countries, the overall stillbirth rate was high at 28.9/1000 (range 13.9 to 56.5/1000) in 2010 and 2013 [49]. Since then, there has been a reduction to 2.4–5.8/1000 in HIC and 5.6–17.9/1000 in LMIC, respectively, from the countries included in this study during 2019 [47]; this may partly be because of global initiatives such as Every Newborn Action Plan and Millennium Developmental Goals (United Nations) [50]. 

Our meta-analysis showed higher stillbirth rates but mostly in LMIC, which are above the Every Newborn Action Plan (ENAP) [51,52] target of 12/1000 or fewer by 2030. However, the contribution from LMIC regions such as Nepal [9] with increased SB rates suggests that regional variations are a result of access to high-quality care; this could be a feature in poor resource areas where the pandemic has affected or disrupted pregnant women’s care. 

The PAN COVID study [53] suggested no difference in SB rates in COVID-19-affected pregnancies. Similarly, an analysis from Spain showed no difference in stillbirths during and before the pandemic period [11]. However, the SB rate from other HIC groups, such as the UK [6], showed an increase in stillbirths during the pandemic compared to the 2019 stillbirth data from the Global Health Observatory data repository [47]. This finding is similar to Hospital Episode Statistics (HES) data and the Office for National Statistics (ONS) data from England. [12] Reported SB rates from LMIC [3] in 2019 were 13.9/1000 (India) [5,6,7,8,9,10,11,12,13,14,15,16,17,18,19,20,21,22,23,24,25,26,27,28,29,30,31,32,33,34,35,36,37] and 7.1/1000 (Peru) [47]. Our meta-analysis shows that these rates have increased considerably to 58 and 32–40/1000 in both India and Peru, respectively, which is represented in our subgroup analysis. 

Comparing the SB in concurrent COVID-19 and non-COVID-19 pregnancies showed an increased SB rate. However, these studies do not provide an accurate representation of the non-COVID-19 group as only the COVID-19 group women tested positive for COVID-19, and therefore, the non-COVID-19 category may have included non-tested, asymptomatic, or mild COVID-19 pregnancies that may have fetal implication. We, therefore, explored the subsequent outcome below to understand the stillbirth difference in a completely different dimension with a population study comparing the pandemic with the pre-pandemic group.

In our pre and post-pandemic period comparative meta-analysis, there was no statistically significant increase in the SB rate during the pandemic period compared to the non-pandemic period. When we looked at the economic income subgroups, there was no statistically significant difference in SB in either the HIC or the LMIC groups. 

It is possible that during the COVID-19 pandemic, the diversion of resources (doctors and premises) towards the prevention and treatment of COVID-19 resulted in the neglect of maternity services. This neglect might have produced a deficiency in care in both HIC and LMIC that has resulted in an increase in SB in some of the reported individual studies. Furthermore, pregnant women might be reluctant to access hospitals for fear of becoming infected and ignoring or forgetting to report adverse pregnancy symptoms, such as a small antepartum hemorrhage or reduced fetal movements.

In the individual studies [27,31,40,54,55] that presented data on the causes of stillbirths, there was no clear evidence to suggest that COVID-19 increased the risk of SB unless there was significant maternal hypoxic or a terminal event that might lead to fetal compromise and intra-uterine fetal demise [29] a fact supported by the findings in the UKOSS data [40] highlighting the need for further information to evaluate the likely impact of significant hypoxia on possible SB rates [40].

Global health focus on stillbirth is ongoing, and there is a need to continue to investigate and identify the causes [56], especially in LMIC [57]. The causes in countries where SB rates continue to be high could be multifactorial, with varied factors such as cut-off points for reporting stillbirths, poverty, education, and maternal diseases, such as syphilis and HIV [57].

Our meta-analysis was not able to identify possible factors accounting for the increase in stillbirths; however, there are ongoing studies that may provide further information on the relationship between COVID-19 and stillbirths [58,59,60,61]. Although our analysis was on stillbirth rates during the pandemic, this cannot be seen in isolation. Factors that are likely to influence stillbirth rates include the type of infection (severity and infectivity of the variant), maternal co-morbidities, and mortality. In a study by Incognito et al. [62], it was shown that infection with the Delta variant of SARS-CoV-2 was associated with a lower APGAR score in newborns and a higher incidence of adverse outcomes (notably preterm birth, fetal growth restriction, and small for gestational age—all of which will increase stillbirth and perinatal mortality rates). In a meta-analysis, Chmielewska et al. [5] showed that global maternal and fetal outcomes worsened during the COVID-19 pandemic with an increase in maternal mortality. These findings were similar to those from the systematic review by La Verde et al. [63], who concluded that COVID-19 with at least one co-morbidity increases the risk of intensive care and maternal mortality. Taken together, these and many studies confirm that women with severe COVID-19 have increased morbidity and mortality and, as would be expected, have a high stillbirth rate.

### Strengths and Limitations

The strength of this meta-analysis is that we included 29 studies, making it the largest on this topic. To better understand changes in stillbirths, we included a comparison of COVID-19 and non-COVID-19 pregnancies, as well as population stillbirths. We included all the relevant published studies for this meta-analysis. The main limitations include the fact that (a) only observational data were analyzed, (b) we did not have data from prospectively collected databases like that in the USA, (c) the data analyzed were heterogeneous, which might have undoubtedly affected the overall results, and (d) the time frame for the analysis might not have allowed us to fully capture the long-term consequences of the pandemic on stillbirth rates. Further research is therefore crucial to deal with these limitations. 

## 5. Conclusions

This meta-analysis shows an overall increase in stillbirths in pregnant women with COVID-19 and predominantly in LMIC. However, when population SB rates were compared between the pre-pandemic and pandemic periods, there was no increase. These findings suggest that while current attention to ensuring that SB rates are unaffected by the pandemic worldwide continues, greater focus must remain on LMIC to ensure the provision of adequate healthcare access during the pandemic while at the same time continuing to investigate all causes of stillbirth and understanding the contribution of the pandemic to regional variations in stillbirths. Such a focus must include concerted efforts to increase vaccination of pregnant women and those in their reproductive years; this must include education to address the problems of vaccine hesitancy, most of which are fueled by misinformation [64]. 

## Data Availability

Not applicable.

## Supplementary Materials

The following supporting information can be downloaded at: https://www.mdpi.com/article/10.3390/jcm12237219/s1, Supplementary Materials S1: title: Search Terms and Strategy; Supplementary Materials S2: title: Excluded studies and reasons for exclusion; Supplementary Materials S3: title: Funnel plotof the precision reates of thje includd studies.
