# Supplementary material for: A Meta-Analysis of the Global Stillbirth Rates during the COVID-19 Pandemic
Source: J Clin Med. 2023 Nov 21;12(23):7219. doi: 10.3390/jcm12237219 (PMC10707675; doi:10.3390/jcm12237219)
Supplement: Supplementary file 1 [file jcm-12-07219-s001.zip › jcm-2670003-supplementary.pdf]

Supplementary Materials S1

Search Strategy:

#	Searches	Results
1	covid-19.ab,ti.	94085
2	covid 19.ab,ti.	94085
3	coronavirus.ab,ti.	49462
4	SARS-COV-2.ab,ti.	30029
5	SARS-Cov2.ab,ti.	1240
6	COVID-19/	62521
7	("stillbirth's" or "stillbirthed" or "stillbirthing" or "still-birth" or "still-births" or "fetus death" or "still born" or "still birth").ab,ti.	6493
8	intrauterine fetal death.mp.	1546
9	intrauterine fetal demise.mp.	556
10	stillbirth.mp. or Stillbirth/	11264
11	1 or 2 or 3 or 4 or 5 or 6	119941
12	7 or 8 or 9 or 10	17168
13	11 and 12	93

Database(s): **Embase** 1974 to 2021 Week 09
Search Strategy:

#	Searches	Results
1	covid-19.ab,ti.	91661
2	covid 19.ab,ti.	91661
3	coronavirus.ab,ti.	48737
4	SARS-COV-2.ab,ti.	28780
5	SARS-Cov2.ab,ti.	1455

6	COVID-19/	92
7	("stillbirth's" or "stillbirthed" or "stillbirthing" or "still-birth" or "still-births" or "fetus death" or "still born" or "still birth").ab,ti.	8889
8	intrauterine fetal death.mp.	2206
9	intrauterine fetal demise.mp.	1108
10	stillbirth.mp. or Stillbirth/	21917
11	1 or 2 or 3 or 4 or 5 or 6	115288
12	7 or 8 or 9 or 10	27684
13	11 and 12	127

CENTRAL

Search Name: covid 19 and stillbirth

Date Run: 06/03/2021 16:54:53

Comment:

ID Search Hits

#1 (covid-19):ti,ab,kw (Word variations have been searched) 4188

#2 (covid 19):ti,ab,kw (Word variations have been searched) 4195

#3 (coronavirus):ti,ab,kw (Word variations have been searched) 2515

#4 (SARS-CoV):ti,ab,kw (Word variations have been searched) 1672

#5 (SARS-CoV2):ti,ab,kw (Word variations have been searched) 172

#6 (SARS-CoV-2):ti,ab,kw (Word variations have been searched) 1635

#7 {OR #1-#6} 4479

#8 stillbirth* 1352

#9 MeSH descriptor: [Stillbirth] explode all trees 132

#10 intrauterine fetal demise 66

#11 intrauterine fetal death 607

#12 ("stillbirth's" or "stillbirthed" or "stillbirthing" or "still-birth" or "still-births" or "fetus death" or "still born" or "still birth"):ti,ab 778

#13 {OR #8-#12} 1859

#14 #7 AND #13 4

WOS

Search History

All Databases Web of Science Core Collection BIOSIS Citation Index BIOSIS Previews Data Citation Index KCI-Korean Journal Database MEDLINE® Russian Science Citation Index SciELO Citation Index Zoological Record Learn More Web of Science Core Collection

Search History

Set	Results	Save History / Create AlertOpen Saved History	Edit Sets	Combine Sets<br>AND OR<br>Combine	Delete Sets<br>Select All<br>Delete
# 5	68	#4 AND #1<br>*Indexes=SCI-EXPANDED, SSCI, A&HCI, CPCI-S, CPCI-SSH, BKCI-S, BKCI-SSH, ESCI Timespan=All years*	Edit		
# 4	15,321	#3 OR #2<br>*Indexes=SCI-EXPANDED, SSCI, A&HCI, CPCI-S, CPCI-SSH, BKCI-S, BKCI-SSH, ESCI Timespan=All years*	Edit		
# 3	14,598	TS=("intrauterine fetal death" OR "intrauterine fetal demise" OR stillbirth)<br>*Indexes=SCI-EXPANDED, SSCI, A&HCI, CPCI-S, CPCI-SSH, BKCI-S, BKCI-SSH, ESCI Timespan=All years*	Edit		

# 2	861	TS=("stillbirth's" or "stillbirthed" or "stillbirthing" or "still-birth" or "still-births" or "fetus death" or "still born" or "still birth")<br>*Indexes=SCI-EXPANDED, SSCI, A&HCI, CPCI-S, CPCI-SSH, BKCI-S, BKCI-SSH, ESCI Timespan=All years*	Edit
# 1	110,810	TS=(covid-19 OR covid19 OR coronavirus OR sars-cov OR sars-cov2 OR sars-cov-2)<br>*Indexes=SCI-EXPANDED, SSCI, A&HCI, CPCI-S, CPCI-SSH, BKCI-S, BKCI-SSH, ESCI Timespan=All years*	Edit

Medline-93

Embase-127

CENTRAL-4

WOS-68

Supplementary Materials S2
Excluded studies with reason and references.

No	Study name	Reason for exclusion
1	Ghayda RA[1]	Systematic review
2	Amaral WN[2]	Systematic review
3	Kasraeian M[3]	Systematic review and meta-analysis
4	Soheili M[4]	Comprehensive evidence synthesis and meta-analysis
5	Papapanou M[5]	Overview of systematic reviews
6	Trippella G[6]	Systematic review
7	Yang Z[7]	Systematic review
8	Juan J[8]	Systematic review
9	Turan O[9]	Systematic review
10	Kotlar B[10]	Scoping review
11	Neef V[11]	Review and meta-analysis
12	Della Gatta AN[12]	Systematic review
13	Di Toro FD[13]	Systematic review and meta-analysis
14	Banaei M[14]	Systematic review
15	Elshafeey F[15]	Scoping review
16	Chi H[16]	Systematic review
17	Bellos I[17]	Meta-analysis
18	Dubey P[18]	Systematic review and meta-analysis
19	Celotto S[19]	Umbrella review
20	Pettirosso E[20]	Review
21	Zaigham M[21]	Systematic review
22	Han Y[22]	Systematic review

23	Yoon SH[23]	Systematic review
24	Rodrigues C[24]	Systematic review
25	Chamseddine RS[25]	Systematic review
26	Dube R[26]	Systematic review
27	Medeiros KS[27]	Systematic review and meta-analysis
28	Chmielewska B[28]	Systematic review and meta-analysis
29	Kakodkar P[29]	Comprehensive literature review
30	Hasnain M[30]	Narrative review
31	Lambelet V[31]	Review
32	Simsek Y[32]	Narrative review
33	Richtmann R[33]	Data not extractable
34	Mullins E[34]	Data not extractable
35	Pirjani R[35]	Data not extractable
36	Martinez-Portilla R[36]	Data not extractable
37	Verma S[37]	Data overlap
38	Pierce-Williams RAM[38]	Data overlap
39	Khalil A[39]	Data overlap
40	Molteni E[40]	Multinational overlap
41	Di Mascio D[41]	Multinational overlap
42	Kumar M[42]	Case-control study

References

1. Abou Ghayda R, Li H, Lee KH, et al. COVID-19 and Adverse Pregnancy Outcome: A Systematic Review of 104 Cases. *J Clin Med*. 2020;9(11):3441. doi:10.3390/jcm9113441
2. Amaral WN do, Moraes CL de, Rodrigues AP dos S, Noll M, Arruda JT, Mendonça CR. Maternal Coronavirus Infections and Neonates Born to Mothers with SARS-CoV-2: A Systematic Review. *Healthcare*. 2020;8(4):511. doi:10.3390/healthcare8040511
3. Kasraeian M, Zare M, Vafaei H, et al. COVID-19 pneumonia and pregnancy; a systematic review and meta-analysis. *J Matern Neonatal Med*. Published online 2020. doi:10.1080/14767058.2020.1763952
4. Soheili M, Moradi G, Baradaran HR, Soheili M, Mokhtari MM, Moradi Y. Clinical manifestation and maternal complications and neonatal outcomes in pregnant women with COVID-19: a comprehensive evidence synthesis and meta-analysis. *J Matern Neonatal Med*. Published online 2021. doi:10.1080/14767058.2021.1888923
5. Papapanou M, Papaioannou M, Petta A, et al. Maternal and Neonatal Characteristics and Outcomes of COVID-19 in Pregnancy: An Overview of Systematic Reviews. *Int J Environ Res Public Health*. 2021;18(2):596. doi:10.3390/ijerph18020596
6. Trippella G, Ciarcià M, Ferrari M, et al. COVID-19 in Pregnant Women and Neonates: A Systematic Review of the Literature with Quality Assessment of the Studies. *Pathogens*. 2020;9(6):485. doi:10.3390/pathogens9060485
7. Yang Z, Wang M, Zhu Z, Liu Y. Coronavirus disease 2019 (COVID-19) and pregnancy: a systematic review. *J Matern Neonatal Med*. Published online April 30, 2020:1-4. doi:10.1080/14767058.2020.1759541
8. Juan J, Gil MM, Rong Z, Zhang Y, Yang H, Poon LC. Effect of coronavirus disease 2019 (COVID-19) on maternal, perinatal and neonatal outcome: systematic review. *Ultrasound Obstet Gynecol*. 2020;56(1):15-27. doi:10.1002/uog.22088
9. Turan O, Hakim A, Dashraath P, Jeslyn WJL, Wright A, Abdul-Kadir R. Clinical characteristics, prognostic factors, and maternal and neonatal outcomes of SARS-CoV-2 infection among hospitalized pregnant women: A systematic review. *Int J Gynecol Obstet*. 2020;151(1):7-16. doi:10.1002/ijgo.13329
10. Kotlar B, Gerson E, Petrillo S, Langer A, Tiemeier H. The impact of the COVID-19 pandemic on

maternal and perinatal health: a scoping review. *Reprod Health*. 2021;18(1):10. doi:10.1186/s12978-021-01070-6

11. Neef V, Buxmann H, Rabenau HF, Zacharowski K, Raimann FJ. Characterization of neonates born to mothers with SARS-CoV-2 infection: Review and meta-analysis. *Pediatr Neonatol*. 2021;62(1):11-20. doi:10.1016/j.pedneo.2020.10.001
12. Della Gatta AN, Rizzo R, Pilu G, Simonazzi G. Coronavirus disease 2019 during pregnancy: a systematic review of reported cases. *Am J Obstet Gynecol*. 2020;223(1):36-41. doi:10.1016/j.ajog.2020.04.013
13. Di Toro F, Gjoka M, Di Lorenzo G, et al. Impact of COVID-19 on maternal and neonatal outcomes: a systematic review and meta-analysis. *Clin Microbiol Infect*. 2021;27(1):36-46. doi:10.1016/j.cmi.2020.10.007
14. Banaei M, Ghasemi V, Saei Ghare Naz M, et al. Obstetrics and neonatal outcomes in pregnant women with covid-19: A systematic review. *Iran J Public Health*. 2020;49(Supple 1):38-47. doi:10.18502/ijph.v49is1.3668
15. Elshafeey F, Magdi R, Hindi N, et al. A systematic scoping review of COVID-19 during pregnancy and childbirth. *Int J Gynecol Obstet*. 2020;150(1):47-52. doi:10.1002/ijgo.13182
16. Chi H, Chiu NC, Tai YL, et al. Clinical features of neonates born to mothers with coronavirus disease-2019: A systematic review of 105 neonates. *J Microbiol Immunol Infect*. 2020;54(1):69. doi:10.1016/j.jmii.2020.07.024
17. Bellos I, Pandita A, Panza R. Maternal and perinatal outcomes in pregnant women infected by SARS-CoV-2: A meta-analysis. *Eur J Obstet Gynecol Reprod Biol*. 2021;256:194-204. doi:10.1016/j.ejogrb.2020.11.038
18. Dubey P, Reddy SY, Manuel S, Dwivedi AK. Maternal and neonatal characteristics and outcomes among COVID-19 infected women: An updated systematic review and meta-analysis. *Eur J Obstet Gynecol Reprod Biol*. 2020;252:490-501. doi:10.1016/j.ejogrb.2020.07.034
19. Celotto S, Veronese N, Barbagallo M, et al. An umbrella review of systematic reviews with meta-analyses evaluating positive and negative outcomes of Hydroxychloroquine and chloroquine therapy. *Int J Infect Dis*. 2021;103:599-606. doi:10.1016/j.ijid.2020.12.018
20. Pettirosso E, Giles M, Cole S, Rees M. COVID-19 and pregnancy: A review of clinical characteristics, obstetric outcomes and vertical transmission. *Aust New Zeal J Obstet Gynaecol*. 2020;60(5):640-659. doi:10.1111/ajo.13204
21. Zaigham M, Andersson O. Maternal and perinatal outcomes with COVID-19: A systematic review of 108 pregnancies. *Acta Obstet Gynecol Scand*. 2020;99(7):823-829. doi:10.1111/aogs.13867
22. Han Y, Ma H, Suo M, et al. Clinical manifestation, outcomes in pregnant women with COVID-19 and the possibility of vertical transmission: A systematic review of the current data. *J Perinat Med*. 2020;48(9):912-924. doi:10.1515/jpm-2020-0431
23. Yoon SH, Kang JM, Ahn JG. Clinical outcomes of 201 neonates born to mothers with COVID-19: A systematic review. *Eur Rev Med Pharmacol Sci*. 2020;24(14):7804-7815. doi:10.26355/eurrev_202007_22285
24. Rodrigues C, Baía I, Domingues R, Barros H. Pregnancy and Breastfeeding During COVID-19 Pandemic: A Systematic Review of Published Pregnancy Cases. *Front Public Heal*. 2020;8:558144. doi:10.3389/fpubh.2020.558144
25. Chamseddine RS, Wahbeh F, Chervenak F, Salomon LJ, Ahmed B, Rafii A. Pregnancy and Neonatal Outcomes in SARS-CoV-2 Infection: A Systematic Review. Marozio L, ed. *J Pregnancy*. 2020;2020:1-7. doi:10.1155/2020/4592450
26. Dube R, Kar SS. COVID-19 in pregnancy: The foetal perspective- A systematic review. *BMJ Paediatr Open*. 2020;4(1). doi:10.1136/bmjpo-2020-000859
27. Medeiros KS, Sarmento ACA, Martins ES, Costa APF, Eleutério J, Gonçalves AK. Impact of SARS-CoV-2 (COVID-19) on pregnancy: A systematic review and meta-analysis protocol. *BMJ*

Open. 2020;10(11):39933. doi:10.1136/bmjopen-2020-039933
28. Chmielewska B, Barratt I, Townsend R, et al. Effects of the COVID-19 pandemic on maternal and perinatal outcomes: a systematic review and meta-analysis. *Lancet Glob Heal*. 2021;0(0). doi:10.1016/S2214-109X(21)00079-6
29. Kakodkar P, Kaka N, Baig M. A Comprehensive Literature Review on the Clinical Presentation, and Management of the Pandemic Coronavirus Disease 2019 (COVID-19). *Cureus*. 2020;12(4):7560. doi:10.7759/cureus.7560
30. Hasnain M, Pasha MF, Ghani I, Budiarto R. Protection challenges of pregnant women against vertical transmission during COVID-19 epidemic: A narrative review. *Am J Infect Control*. 2020;48(12):1516-1519. doi:10.1016/j.ajic.2020.06.206
31. Lambelet V, Vouga M, Pomar L, et al. SARS-CoV-2 in the context of past coronaviruses epidemics: Consideration for prenatal care. *Prenat Diagn*. 2020;40(13):1641-1654. doi:10.1002/pd.5759
32. Simsek Y, Ciplak B, Songur S, Kara M, Karahocagil MK. Maternal and fetal outcomes of COVID-19, SARS, and MERS: A narrative review on the current knowledge. *Eur Rev Med Pharmacol Sci*. 2020;24(18):9748-9752. doi:10.26355/eurrev_202009_23068
33. Richtmann R, Torloni MR, Oyamada Otani AR, et al. Fetal deaths in pregnancies with SARS-CoV-2 infection in Brazil: A case series. *Case Reports Women's Heal*. 2020;27:e00243. doi:10.1016/j.crwh.2020.e00243
34. Mullins E, Hudak ML, Banerjee J, et al. Pregnancy and neonatal outcomes of <scp>COVID</scp> -19: co-reporting of common outcomes from <scp>PAN-COVID</scp> and <scp>AAP SONPM</scp> registries. *Ultrasound Obstet Gynecol*. Published online February 23, 2021:uog.23619. doi:10.1002/uog.23619
35. Pirjani R, Hosseini R, Soori T, et al. Maternal and neonatal outcomes in COVID-19 infected pregnancies: a prospective cohort study. *J Travel Med*. 2020;27(7). doi:10.1093/jtm/taaa158
36. Martinez-Portilla RJ, Sotiriadis A, Chatzakis C, et al. Pregnant women with SARS-CoV-2 infection are at higher risk of death and pneumonia: propensity score matched analysis of a nationwide prospective cohort (COV19Mx). *Ultrasound Obstet Gynecol*. 2021;57(2):224-231. doi:10.1002/uog.23575
37. Verma S, Bradshaw C, Freda Auyeung N, et al. Outcomes of Maternal-Newborn Dyads After Maternal SARS-CoV-2. doi:10.1542/peds.2020-005637
38. Pierce-Williams RAM, Burd J, Felder L, et al. Clinical course of severe and critical coronavirus disease 2019 in hospitalized pregnancies: a United States cohort study. *Am J Obstet Gynecol MFM*. 2020;2(3):100134. doi:10.1016/j.ajogmf.2020.100134
39. Khalil A, Dadelszen P von, Draycott T, Ugwumadu A, O'Brien P, Magee L. Change in the Incidence of Stillbirth and Preterm Delivery During the COVID-19 Pandemic. *JAMA - J Am Med Assoc*. Published online 2020. doi:10.1001/jama.2020.12746
40. Molteni E, Astley CM, Ma W, et al. SARS-CoV-2 (COVID-19) infection in pregnant women: Characterization of symptoms and syndromes predictive of disease and severity through real-time, remote participatory epidemiology. *medRxiv*. Published online October 14, 2020. doi:10.1101/2020.08.17.20161760
41. Di Mascio D, Sen C, Saccone G, et al. Risk factors associated with adverse fetal outcomes in pregnancies affected by Coronavirus disease 2019 (COVID-19): a secondary analysis of the WAPM study on COVID-19. *J Perinat Med*. 2020;48(9):950-958. doi:10.1515/jpm-2020-0355
42. Kumar M, Puri M, Yadav R, et al. Stillbirths and the COVID-19 pandemic: Looking beyond SARS-CoV-2 infection. *Int J Gynecol Obstet*. 2021;153(1):76-82. doi:10.1002/ijgo.13564

Supplementary Materials S3 (Funnel plot)

Funnel plot for outcome 1: SB rate in pregnant women with COVID-19 (overall)

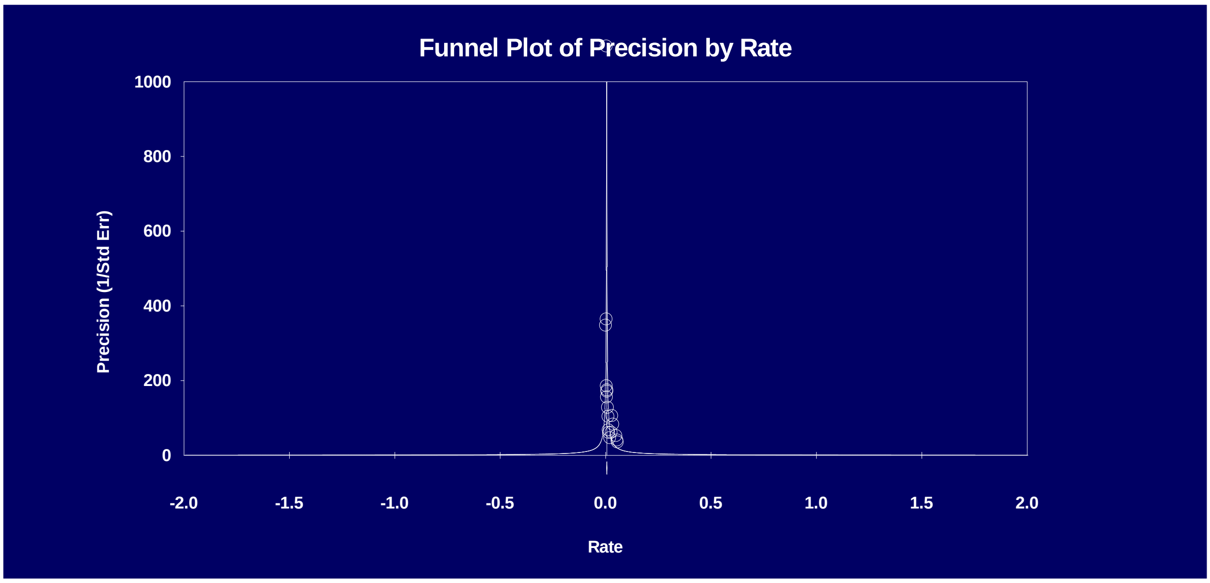

Funnel plot for outcome 2: SB rates in pregnant women with and without COVID-19 at the same time period.

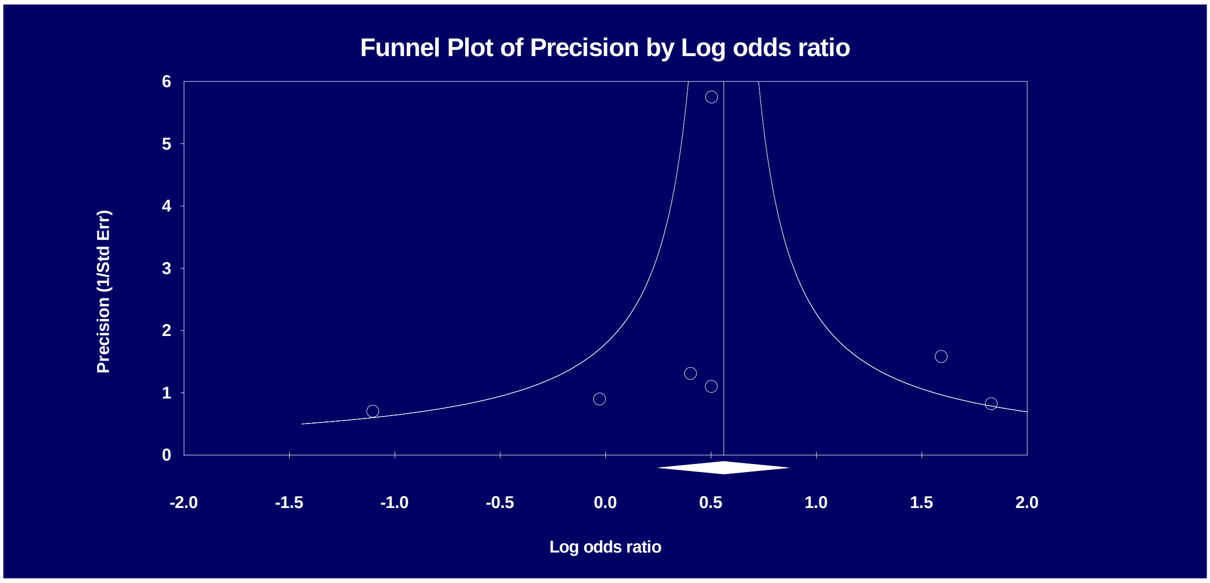

Funnel plot for outcome 3: Population SB rates in pre-pandemic and pandemic periods.

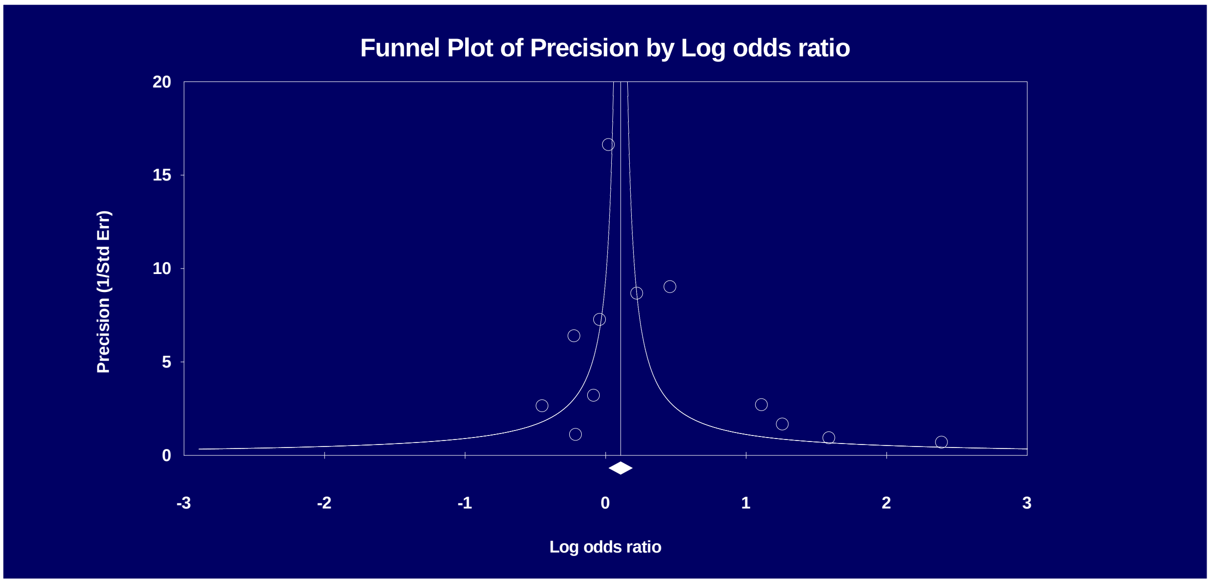

Funnel Plot of Precision by Log odds ratio
Precision (1/Std Err)
20
15
10
5
0
-3
-2
-1
0
1
2
3
Log odds ratio
